# One-Pot Synthesis of Amine-Functionalized Nano-Silica via Sol-Gel Assisted by Reverse Micelle Microemulsion for Environmental Application

**DOI:** 10.3390/nano12060947

**Published:** 2022-03-13

**Authors:** Enshirah Da’na, Wafa Shamsan Al-Arjan, Sukainah Al-Saeed, Mohamed Ramadan El-Aassar

**Affiliations:** 1 Biomedical Engineering Department, College of Engineering, King Faisal University, P.O. Box 400, Al-Ahsa 31982, Saudi Arabia; 2 Department of Chemistry, College of Science, King Faisal University, P.O. Box 400, Al-Ahsa 31982, Saudi Arabia; walarjan@kfu.edu.sa (W.S.A.-A.); sukalsaeed91@hotmail.com (S.A.-S.); 3 Department of Chemistry, College of Science, Jouf University, Sakaka 2014, Saudi Arabia; mrelaassar@ju.edu.sa

**Keywords:** nanocomposite, microemulsion, heavy metals, reverse micelle, nano-silica, one-pot synthesis

## Abstract

Amine modified nano-silica was prepared via a one-pot route and under very mild conditions in water in oil microemulsion with a non-ionic surfactant to study the effect of changing the amount of N-[3-(Trimethoxysilyl)propyl]ethylenediamine (DA) added to the synthesis mixture on the characteristics of the obtained nanocomposite such as morphology, crystallinity, surface charge, particle size, surface area, and accordingly the effect of all of these factors on the efficiency of the nanocomposite for the removal of heavy metal ions, namely zinc, from aqueous solutions. XRD, SEM, TGA, BET, DLS, FTIR, and pH_0_ analysis were performed for samples and the results showed a strong effect for the amount of DA added to the synthesis mixture on the characteristics of the obtained nanocomposites. It was found that increasing the amount of DA added to the synthesis mixture increased the pH_0_, hydrodynamic particle size obtained by dynamic light scattering analysis, and the particle size obtained by SEM. Sample prepared without the addition of DA (SNP) and the samples prepared with 1.5 mL of DA (SNP-1.5DA) showed a better adsorption performance compared to the samples prepared with 0.5 and 1.0 mL of DA (SNP-0.5DA and SNP-1.0DA, respectively). The main factor affecting the adsorption efficiency was found to be the available surface area for each nanocomposite, which was directly related to the degree of crystallinity as obtained by XRD analysis.

## 1. Introduction

Silica nanoparticles are one of the most known stable, biocompatible, inert, and non-toxic materials [1,2]. Furthermore, its silanol-rich surface makes it a perfect support for a wide range of applications via anchoring different functional groups [2,3,4]. The synthesis of uniform silica nanoparticles with a particle size in the range 100–1000 nm started by hydrolysis condensation of TEOS from aqueous solution catalyzed with ammonium in the early of 1960 [5]. Later on, much smaller silica nanoparticles were successfully prepared via microemulsion technique through the dispersion of water phase within a continuous oil phase with the aid of a suitable surfactant at the interface as stabilizing agent [5]. The micro water droplets encapsulated within the surfactant core act as a microreactor for the hydrolysis condensation of TEOS into nano-silica with excellent control over the particle size distribution via controlling the size of the droplets. Further improvement was achieved by adding a co-surfactant to increase the stability of the produced micro emulsion [5].

Next, researchers focused on the ability to control and improve the surface properties of the nano-silica such as electrical and optical [6], mechanical strength [7], hydrophobicity, and hydrophilicity [8]. It was reported that increasing hydrophilicity of the surface enhances the water flux and accordingly facilitates reaching the active sites on the surface by the target components in the aqueous phase [8]. One effective way of improving hydrophilicity is to anchor the surface with amino groups (SiO_2_–NH_2_). In addition to this, it is reported that amino groups attached to the surface improve the affinity of the surface to some target molecules such as heavy metal ions, CO_2_, and some biological components [2]. Pandey et al. (2021) anchored 3-aminopropyltrimthoxysilane (APTMS) to the surface of nano-silica to enhance its affinity toward capturing CO_2_ [9], while Zhang et al. (2021) anchored 3-aminopropyl) triethoxysilane (APTES) to prepare an efficient adsorbent to extract heparin [10]. Devlin et al. (2021) prepared enzyme-modified silica nanoparticles to attract staphylococcus aureus and disperse biofilms [11]. Thus, silica nanoparticles have become one of the most attractive materials for different applications such as catalysis, adsorption, chromatography, ceramics, biomedical devices, electronic substances, stabilizers, coating, sensors, and many others [1,2,5].

Accordingly, the demand for specific designs and synthesis of silica nanoparticles has emerged to meet the requirements. All the reported surface modifications of the nano-silica were achieved by additional steps after the synthesis of the bare nano-silica, which consumes more effort, time, and chemicals. Meddeb et al. (2021) grafted β-cyclodextrin on the surface of silica nanoparticles [6]. Wang et al. (2021) followed a post-synthesis procedure to introduce the multi-hydroxyl-containing gemini surfactant to the surface of silica nanoparticles at 70 °C and applied it for the adsorption of methyl orange [12]. Liu et al. (2021) anchored the thymol functionality to the silica nanoparticles by two steps via a simple impregnation method and studied its antimicrobial efficiency [13]. Bamane and Jagtap (2021) followed a two-step technique through grafting of glycidyloxypropyl trimethoxysilane on the surface of nano-silica followed by modifying it with dimethyl propionic acid for self-cleaning coatings applications [8].

High levels of heavy metals in water are toxic to humans, animals, and aquatic systems [14]. Thus, it is vital to remove these ions from wastewater using a variety of methods such as chemical precipitation, membrane filtration, electrochemical technique, ion exchange, and adsorption [15]. Above all, adsorption is very attractive due to its low cost, simple operation and maintenance, and also can be very selective, efficient, and fast if a suitable adsorbent is used for the target pollutant [15]. Zinc will be used in this work due to its extensive usage in metallurgy, power plants, transportation, and construction. Zinc is a major nutrient for the human body at the micro-level of concentration. However, a high intake of zinc may result in stomach cramps, skin irritations, anemia, vomiting, and damage of the pancreas, negatively affecting the immune system and the protein metabolism [14]. Accordingly, the World Health Organization (WHO) has set its limit in drinking water to 3 ppm [16].

The main challenge is to control the morphology, size, and functionality of the materials to improve their performances in the targeted applications [2]. This work is aiming to synthesize amine-modified nano-silica via a simple one-step method under very mild conditions. In addition, this route of introducing amine functionality to the silica nanoparticles is expected to allow achieving high loading of amine that is evenly distributed within the structure, in addition to saving time, effort, and chemicals if the correct ratios of materials were used for the synthesis.

## 2. Materials and Methods

Tetraethylorthosilicate (TEOS) and 98% Methanol were obtained from Merck, Triton X-100, 99.5% cyclohexane, and N-[3-(Trimethoxysilyl)propyl]ethylenediamine (DA) were supplied by Sigma-Aldrich (Missouri, MO, USA).

### 2.1. Synthesis of the Nanocomposite

The microemulsion was prepared via mixing of the surfactant (Triton X-100), co-surfactant (Methanol), and the oil phase (Cyclohexane) according to Table 1 at 700 rpm for 15 min at 20 °C. The pH of the microemulsion was adjusted to a basic medium by adding 2 mL of ammonium hydroxide solution to catalyze the polymerization reaction for the growth of the silica particles. In a separate beaker, the silica precursor (TEOS) was hydrolyzed by mixing it with water for 10 min. After that, the TEOS-water mixture was added to the micro-emulsion and stirred for 10 min. Then, the required amount of N-[3-(Trimethoxysilyl)propyl]ethylenediamine (DA) was added and stirred at 20 °C for 24 h. The microemulsion was then washed with ethanol. The nanoparticles were recovered by filtration, then washed with ethanol to remove the excess of surfactant, co-surfactant, and DA. The resulting nanocomposite was finally dried at 70 °C in a convection oven for 2 h before being stored in a well-sealed container until used.

### 2.2. The pH of Zero Charges (pH_0_)

To measure the pH of zero charges (pH_0_) for the prepared silica nanocomposites, a 0.1 M NaCl solution was used as a background electrolyte. This solution was then divided into 6 beakers. Each beaker was adjusted at different initial pH (pH_i_) within the range 2–12 using HNO_3_, KOH, and Orion 2-star pH meter. Next, 10 mg of each sample was mixed with 20 mL of each NaCl solution at 100 rpm and 20 °C for 24 h to ensure reaching equilibrium. After that, the equilibrium pH (pH_e_) of each sample was measured. A plot of ∆pH versus pH_i_ is used to find the pH_0_, which represents the intercept of the curve with the x-axes.

### 2.3. Preparation of Zinc Ions Solutions

To prepare a 100 ppm Zn^2+^ solution, 455 mg of Zn(NO_3_)_2_.6H_2_O salt was dissolved in double distilled water to make a volume of 1 L. This solution was then successively diluted to get 80, 60, 40, and 20 ppm solutions to be used for the equilibrium test. The pH of all solutions was adjusted at 7.2 using KOH and Orion 2-star pH meter. This pH was used to enhance the surface charge of the adsorbent, as will be discussed later. Furthermore, it was calculated based on the ksp value of the precipitation of Zn(OH)_2_ at 20 °C to ensure that all the Zn^2+^ will be in this ionic form and not precipitated as Zn(OH)_2_.

### 2.4. Equilibrium Test

Each of the four prepared samples was tested to adsorb Zn^2+^ from four different concentrations (100, 80, 60, 40, and 20 ppm). For each experiment, 20 mg of each sample was mixed with 20 mL of each solution and stirred at 70 ppm for 24 h to ensure reaching equilibrium. After that time, the solid adsorbent was separated from the solution by vacuum filtration using Whatman filter papers. The filtrate samples were all analyzed by atomic absorption spectrometer (iCE 3000 Series Atomic Absorption, Quattro S, Missouri, MO, USA) to detect the final concentration of the Zn^2+^ ions that remained in the solution. The adsorbed ions (*q_e_*) and the removal efficiency (*R*) were calculated using Equations (1) and (2):

(1) $$q_{e}=\frac{\left(C_{i}-C_{e}\right)V}{m}\;$$

(2) $$R=\frac{C_{i}-C_{e}}{C_{i}}\times 100$$

where *C_i_* and *C_e_* are the initial and equilibrium Zn^2+^ concentration (mg.L^−1^), respectively. V is the volume of the solution in L, and m is the mass of the adsorbent in g. Langmuir adsorption isotherm was used to fit the equilibrium data via nonlinear regression with the aid of Origin 2020 software using Equation (3):

(3) $$q_{e}=\frac{q_{m}K_{L}C_{e}}{1+K_{L}C_{e}}$$

where *q_m_* and *K_L_* are the equilibrium adsorption capacity (mg.g^−1^) and Langmuir constant (L.mg^−1^), respectively.

### 2.5. Kinetic Test

Each of the four prepared samples was tested to adsorb Zn^2+^ from 40 ppm solutions for different time intervals. For each experiment, 40 mg of each sample was mixed with 100 mL of the solution and stirred at 70 ppm for 2 h. During stirring, a solution sample was taken after different periods of 5, 20, 30, 40, 50, 60, 90, and 120 min and filtered using Whatman filter papers. The filtrate samples were all analyzed by atomic absorption spectrometer (iCE 3000 Series Atomic Absorption, Quattro S, USA) to detect the final concentration of the Zn^2+^ ions that remained in the solution. Pseudo-second-order model was used to fit the kinetic data via nonlinear regression with the aid of origin 2020 software using Equation (4):

(4) $$q_{t}=\frac{K_{P2}q_{e}^{2}t}{1+K_{P2}q_{e}t}$$

where *q_e_* and *K_P_*_2_ are the equilibrium adsorption capacity (mg.g^−1^) and Pseudo-second-order rate constant (g.mg^−1^.min^−1^), respectively.

### 2.6. Characterization

The X-ray diffraction of the samples was performed with XRD-7000 with Cu detector, Shimadzu Company, Japan. The morphological structures of the samples were investigated via scanning electron microscopy FESEM, Thermo Scientific, Quattro S, USA. The thermal stability of the samples was evaluated using thermal gravimetric analysis (TGA-51 Shimadzu Thermogravimetric Analyzers, Japan) within the temperature range of 25–600 °C. The surface analysis was performed with ATR-FT-IR spectrometer (IR–Tracer 100 Fourier Transform Infrared Spectrophotom, Shimadzu, Japan). The nitrogen adsorption-desorption isotherms at 77 K were measured using A NOVA 4200e (Quantachrome Instruments, Missouri, MO, USA). The specific surface area and pore size were further analyzed using the Brunauer–Emmett–Teller (BET) equation. In addition, Pore size distributions were estimated using the isothermic adsorption branch (BJH) Barrett–Joyner–Halenda technique. Dynamic light scattering (DLS) was performed using Cilas, Orléans, France, dual scattering particle size analyzer Nano DS. The sample was prepared by sonication of 10 mg of silica samples in 10 mL of double distilled, deionized water using power sonic 405 at 20 °C without any pH adjustment for 1 h. Dispersion of nanomaterial suspension was performed immersing the tube in a cold-water bath during sonication with an ultrasonic system (power sonic 405).

## 3. Results and Discussion

**Figure 1:**
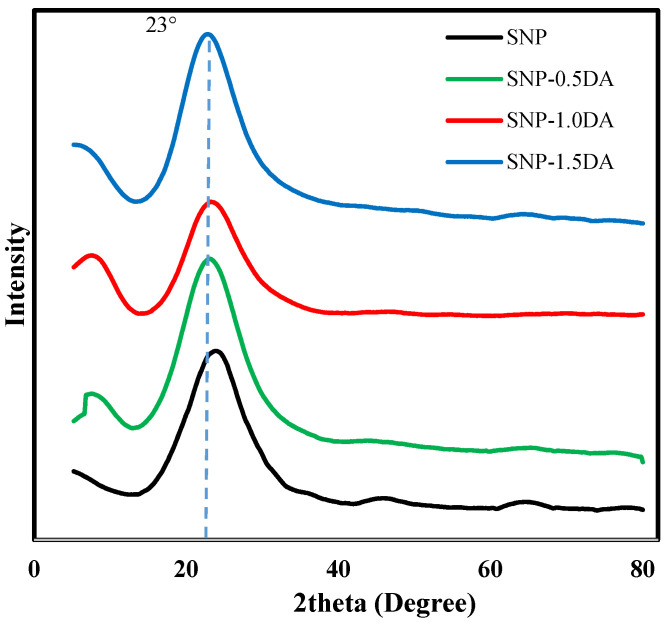
XRD spectra for the four nanocomposites over the 2Ɵ range of 5–80°.

The crystal structure was studied through the powder X-ray diffractometer (λ =  1.54056 nm) at a scanning rate of 0.02 °/s in the 2θ range of 5° to 80°. Figure 1 shows that the XRD pattern of all samples shows a major characteristic broad peak at 2θ = 23° indicating an amorphous nature of the obtained nanocomposites.

**Figure 2:**
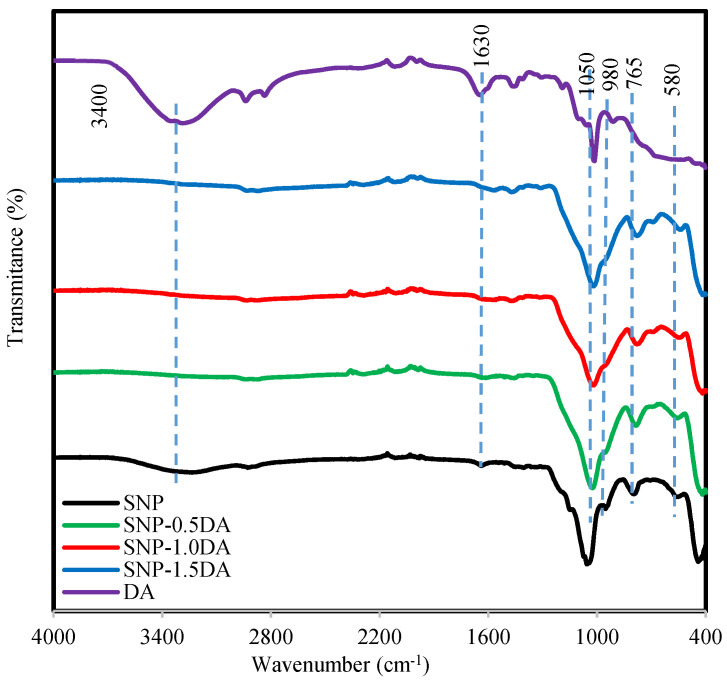
FTIR for the four nanocomposites and the DA over the wavenumber range of 400–4000 cm^−1^.

Figure 2 shows the FTIR for the four nanocomposites and the DA used in the synthesis. SNP and DA spectra show a broad peak around 3400 cm^−1^, which indicates a hydrogen bond due to the existence of hydroxyl (-OH) and (H_2_O) adsorbed on the surface of the nanocomposite [17,18]. The other common peak between SNP and DA spectra is located around 1630 cm^−1^ and is related to the hydroxyl group (-OH) [17]. The disappearance of the broad peak at 3400 cm^−1^ in the SNP-0.5DA, SNP-1.0DA, and SNP-1.5DA indicated that the hydroxyl groups (-OH) have been involved in the reaction with DA during the synthesis process. Similarly, the band at 980 cm^−1^ in the SNP and DA spectra corresponds to the stretching vibration of Si-OH, this band decreased by increasing the amount of DA added to the synthesis mixture of SNP-0.5DA, SNP-1.0DA, and SNP-1.5DA due to the consumption of the silanol groups in the reaction [10]. The peak that appears in all samples at 1050 cm^−1^ is related to the C-O stretch [17]. The peaks around 580 cm^−1^ and 765 cm^−1^ in the four nanocomposites are attributed to the Si-O- Si stretching vibration [7] and bending vibrations [9]. The possible reason for the undetected band at 3400 cm^−1^ of the N-H stretching vibrations is the strong interaction between the terminal NH_2_ of the aminopropyl groups and the unreacted surface hydroxyls on the silica [19]. A similar observation was reported in the literature with no detection of N-H stretching vibrations of amine-modified silica [20,21,22]. The weak band centered at around 1450 cm^−1^ appeared and was assigned to the NH_2_ deformation mode of the amine group [20].

**Figure 3:**
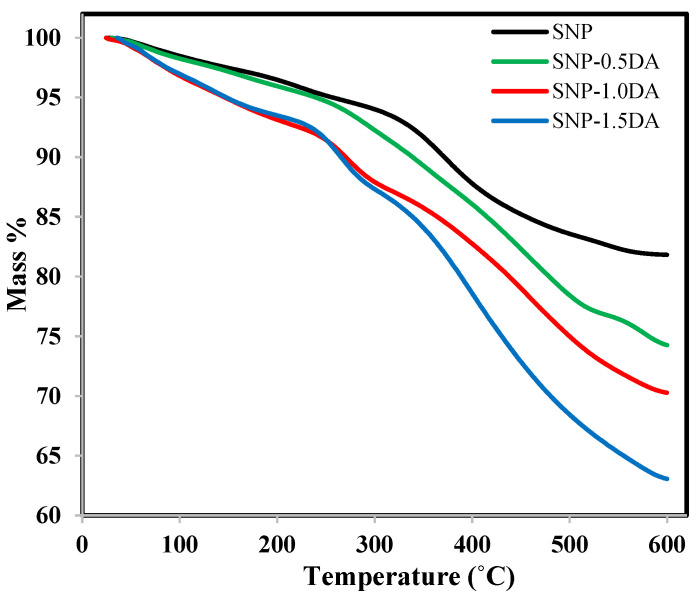
TGA profile for the four nanocomposites over the temperature range of 20–600 °C.

The thermal stability of the nanocomposites was investigated via thermogravimetric analysis (TGA). Figure 3 shows the mass loss of solid for each nanocomposite as a function of temperature. It shows that the four nanocomposites are very stable until 250 °C with a maximum loss that did not exceed 5% for SNP and SNP-0.5DA and 7% for SNP-1.0DA and SNP-1.5DA. The mass loss during this stage is mainly related to the loss of weakly adsorbed molecules on the surface such as N_2_, CO_2_, and H_2_O. As the temperature increased above 250 °C, a clear difference between the four nanocomposites can be noticed. For SNP, the loss in this stage is mainly related to the loss of water strongly attached to the surface or within the pores in addition to the dissociation of any surfactant or co-surfactant residuals within the sample with a total mass loss of 18%. In addition, SNP-0.5DA, SNP-1.0DA, and SNP-1.5DA lost more mass (25%, 30%, and 37%, respectively) due to the decomposition of the amino group, which extends to 600 °C. The higher the amount of DA added to the synthesis mixture, the higher the DA content of the prepared nanocomposite, and accordingly more mass loss was obtained by the TGA.

**Figure 4:**
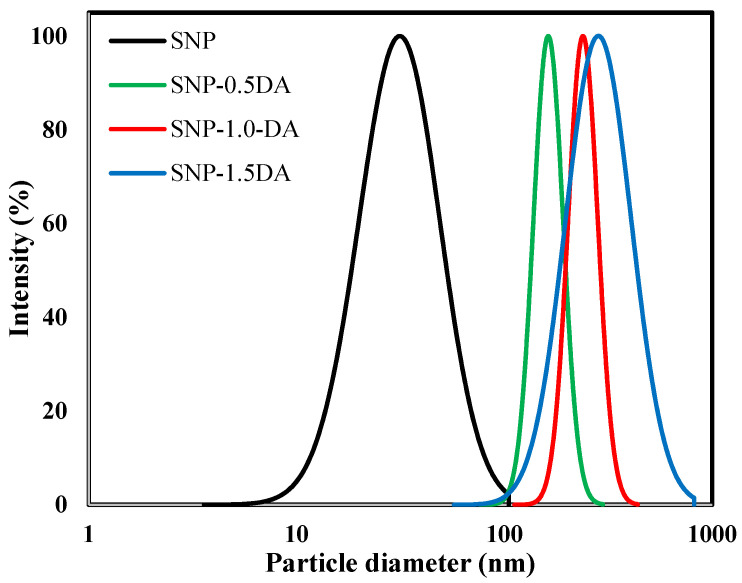
DLS profiles of silica nanocomposites prepared with different amounts of DA (1 mL of silica sample with 10 mL deionized-double distilled water at 20 °C).

Figure 4 shows the results of the dynamic light scattering analysis (DLS). It is obvious that increasing the amount of DA added to the synthesis mixture resulted in larger hydrodynamic particle size with a broader distribution. The center of particle size distribution has been shifted from ≈34 nm for SNP to ≈168, 235, and 295 nm for SNP-0.5DA, SNP-1.0DA, and SNP-1.5DA, respectively, in agreement with the results obtained by SEM to be discussed later. This may be explained as adding the DA to the synthesis mixture increases the condensation of silica resulting in a higher growth rate and thus larger particle size. It is also possible that the higher DA content increases the tendency of the particles to aggregate, as will be discussed later with SEM and BET analysis.

**Figure 5:**
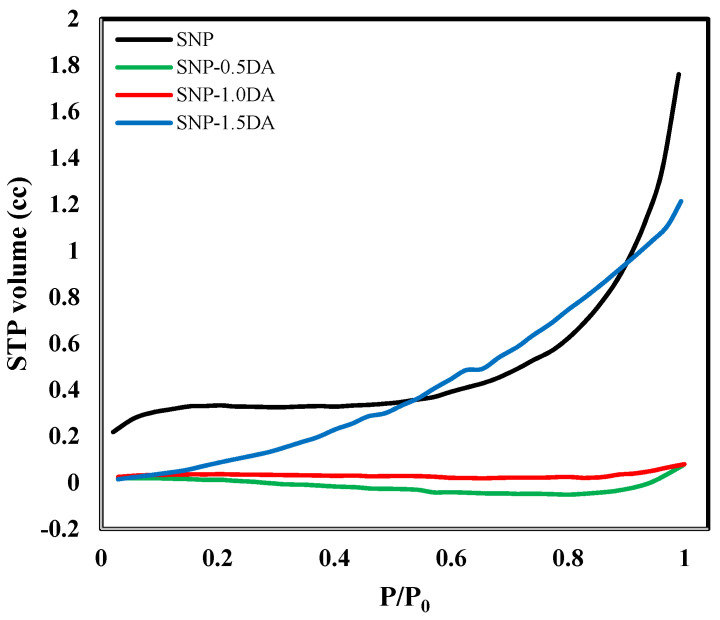
The nitrogen adsorption isotherms of the silica nanocomposites prepared with different amounts of DA.

The N_2_ adsorption profile shown in Figure 5 indicated that the porous structure of the nanocomposites is also significantly affected by the amount of DA used for the preparation (Table 1). The SNP isotherm reveals the presence of mesoporous (2 nm < pores < 50 nm) with the largest surface area (26.95 m^2^g^−1^) and pore volume (0.1823 cm^3^g^−1^), which is related to the amorphous nature and low degree of aggregation for this sample [23]. Adding DA to the synthesis mixture resulted in nonporous structure for both SNP-0.5DA and SNP-1.0DA with extremely small specific surface area and pore volume, as shown in Table 2. On the other hand, nanocomposite prepared with 1.5 mL DA exhibits isotherms with open porous structures (Figure 5) and a specific surface area of 9.836 m^2^g^−1^ and pore volume of 0.0768 cm^3^g^−1^. The drastic decrease in the specific surface area and pore volume of both SNP-0.5DA and SNP-1.0DA is related to the higher degree of aggregation in these two samples, which resulted in the loss of the majority of the pores. The effect of adding DA to the synthesis mixture on the porous structure of the nanocomposites is in agreement with the effect on the adsorption efficiency, to be discussed later since the availability of high surface area with accessible active sites within the pores is one of the most important factors affecting the efficiency of adsorption [24].

**Figure 6:**
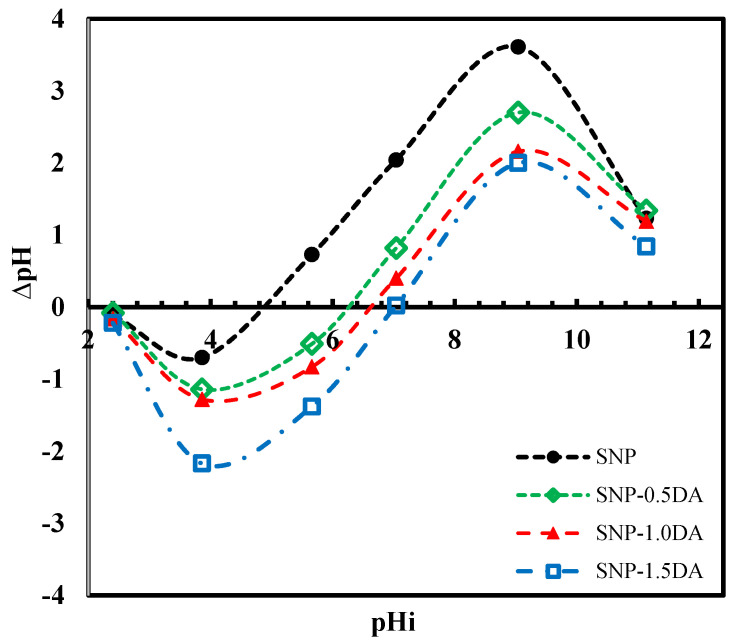
The pH of zero-point charge equilibrium curve for the nanocomposites prepared with different amounts of DA.

The surface charge of SNP-DA is a result of the protonation or deprotonation of surface OH and NH_2_ groups. Accordingly, the pH_0_, which is the pH value of the solution corresponding to zero surface charge, is the dependence of adsorption of Zn^+2^ on the solution pH [25]. Figure 6 shows the ∆pH (pH_i_-pH_e_) versus pHi equilibrium curve. The x-intercepts of these curves represent the pH_0_ of SNP, SNP-0.5DA, SNP-1.0DA, SNP-1.5DA which are 4.9, 6.3, 6.6, and 7.0, respectively, as recorded in Table 2. The pH_0_ of SNP is a higher-than-expected value for SiO_2_ (around 2.5), which may be related to the presence of some surfactant residual within the silica structure since mild extraction was used for the removal of the surfactant rather than calcination. This is supported by the 18% mass loss of SNP at temperature higher than 250 °C (Figure 3). Adding DA to the synthesis mixture resulted in shifting the pH_0_ to a higher value, and also this pH_0_ increased by increasing the amount of DA added. A similar observation about the zero-point charge of the silica and the effect of introducing amine was reported in the literature [26]. The obtained pH_0_ values imply that in aqueous solution with a pH less than pH_0_, but higher than pK_b_ of the DA (around 4), the surface will be rich with ≡SiOH^2+^ species, while at any pH lower than the pK_b_, the SNP-DA surface will bear a more positive charge as a result of the protonation of both OH and NH_2_ groups. Thus, the material will not be an effective Zn^+2^ adsorbent as a result of repulsive forces. On the other hand, the SNP-DA will bear a negative charge if placed in a solution with a pH higher than 7.0 due to the deprotonation of the OH groups. Accordingly, and since the target of this work was to adsorb the Zn^+2^ cations, all the adsorption tests were performed after adjusting the pH at 7.2 to ensure a negatively charged surface and at the same time to prevent the precipitation of Zn(OH)_2_. Ali et al. (2020) prepared thiosemicarbazide-modified nanosilica and reported a pH_ZCP_ of 6.5 [27]. They applied the sample for the adsorption of copper from a solution and reported maximum removal efficiency of 94% at a pH of 7.0.

**Figure 7:**
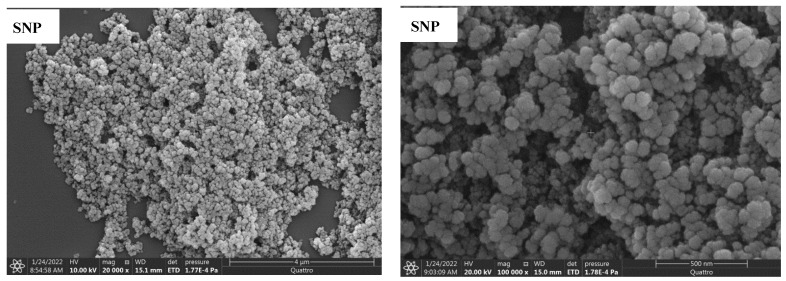

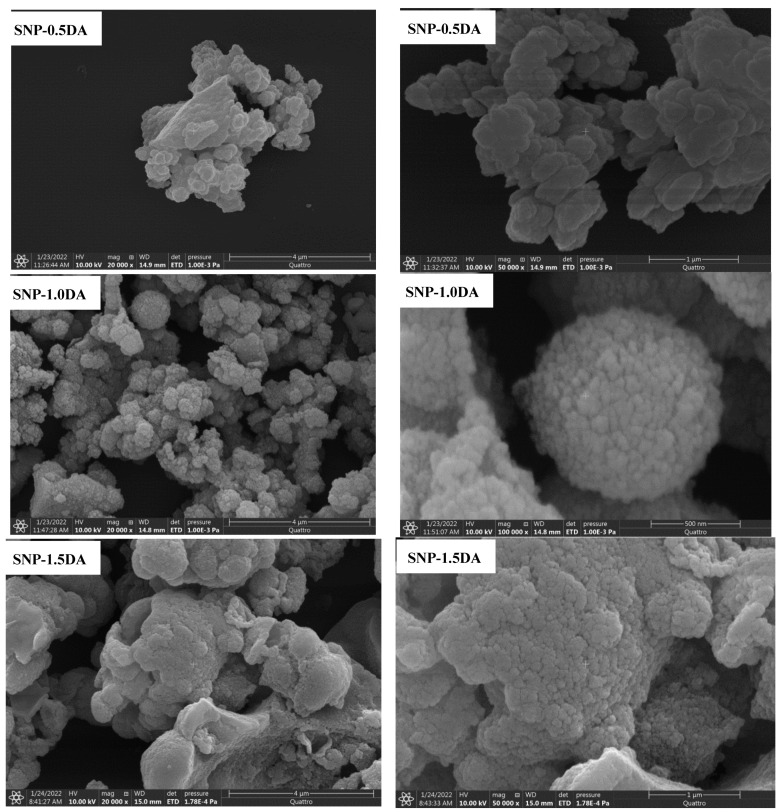
The SEM images of the nanocomposites were prepared with different amounts of DA at two different magnifications.

Figure 7 shows the SEM images for the prepared samples. SNP sample shows almost spherical particles with narrow size distribution and some tendency to agglomeration. After incorporating the DA within the nanocomposite structure, a higher degree of aggregation resulted in irregular particle shapes and wider size distribution, in agreement with the DLS analysis discussed earlier. This higher degree of aggregation obtained after adding DA may be related to the enhanced hydrophilicity of the surface in addition to the tendency to form hydrogen bonds between surface hydroxyl groups and the amine groups attached to the surface. This growth of particle size and aggregation resulted in lower surface areas and pore volume, as obtained by nitrogen adsorption. Accordingly, less surface area will be available for adsorption, as will be next discussed.

**Table 2:** Characteristics of the prepared nanocomposites.

Sample	pH_0_	BET (m^2^g^−1^)	Pore Size PJH (nm)	Pore Volume PJH (cm^3^g^−1^)	TGA Mass Loss (%)	Morphology
SNP	4.9	26.95	25.09	0.182	18.2	Spherical
SNP-0.5DA	6.3	0.848	56.58	0.023	25.7	Irregular
SNP-1.0DA	6.6	3.982	23.14	0.032	29.7	Irregular
SNP-1.5DA	7.0	9.836	15.65	0.077	36.9	Irregular

**Figure 8:**
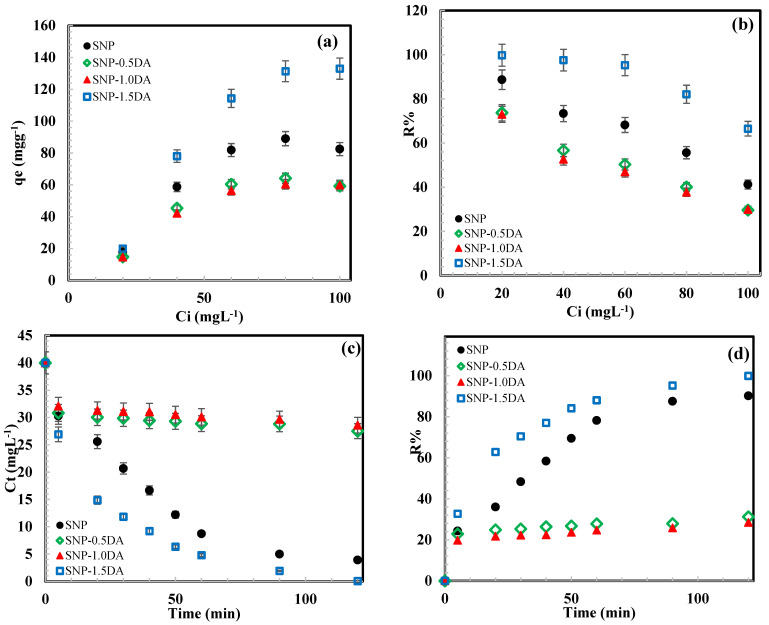

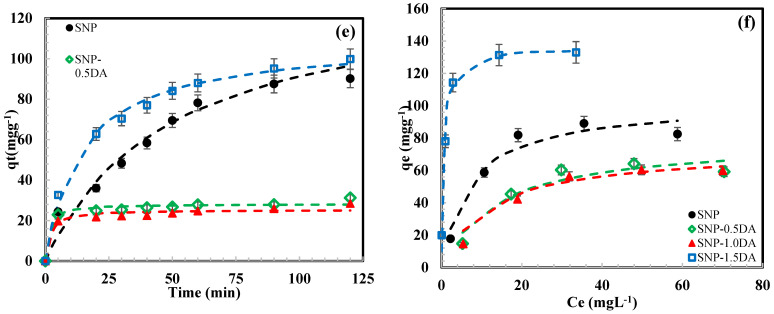
The dependence of equilibrium adsorption capacity on Zn^+2^ initial concentration (**a**), removal efficiency on Zn^+2^ initial concentration (**b**), Zn^+2^ concentration on time (**c**), equilibrium adsorption capacity on Zn^+2^ equilibrium concentration (**d**), adsorption capacity on time (**e**) and removal efficiency on time (**f**).

Figure 8a shows the effect of initial Zn^2+^ concentration on the adsorption capacity that was achieved by each sample. As the initial concentration increase, the driving force for diffusion of ions from the bulk solution to the adsorbent surface increase, and accordingly higher adsorption can be reached till equilibrium is established and all the available adsorption sites are being occupied. Figure 8a shows that the equilibrium adsorption capacities follow the following order SNP-1.5DA > SNP > SNP-0.5DA > SNP-1.0DA. The maximum adsorption capacity was achieved by SNP-1.5DA, which could be attributed to the high surface area and pore size (Table 2), and a surface rich with DA groups that are accessible for Zn^+2^. Samples SNP-0.5DA and SNP-1.0DA have lower adsorption capacity than SNP despite the existence of the DA. This is mainly related to the aggregation that occurred in these samples which resulted in very low accessibility of the majority of the DA groups within the blocked pores. On the other hand, SNP sample showed a better adsorption capacity since it has the highest surface area, and its surface is rich with OH groups as shown by FTIR in Figure 2. These OH groups were deprotonated since the pH of the solution was higher than the pH_0_, and, accordingly, the surface will be negatively charged and attract the positive Zn^+2^ cations.

Figure 8b represents the effect of increasing the initial concentration on the removal efficiency. As the concentration increases the removal efficiency decreases as a result of the saturation of the surface with Zn^+2^. Sample SNP-1.5DA was able to 100% remove Zn^+2^ from a 20-ppm solution, 98% from a 40-ppm solution, and 95% from a 60 ppm solution. While SNP, SNP-0.5DA, and SNP-1.0DA removed 89%, 74%, and 73% from the 20-ppm solution, respectively.

Figure 8c,d show the change of concentration and removal efficiency as a function of time. For samples SNP-0.5DA, and SNP-1.0DA there was a very fast drop in C_t_ and an increase in R% with the equilibrium reached in about 5 min., which is mainly because these samples have a high degree of aggregation, low surface area, and, accordingly, all the accessible adsorption sites (DA groups) are located on the external surface area, so there was no need for diffusion within the porous structure. For the other two samples, three stages are shown, a fast drop in C_t_ and an increase in R% during the first 5 min, which is related to the adsorption achieved by the active sites available on the external surface area of the samples. The second stage (5–90 min) was slower since adsorption is taking place on the active sites within the pores, and thus more time is needed for diffusion. The third stage is the equilibrium stage (after 90 min) where both C_t_ and R% remained constant as a result of the occupation of all active sites. Even though SNP has a higher surface area than SNP-1.5DA, the latter one showed faster kinetic than the SNP. This may be attributed to the effect of DA, which is expected to increase the affinity toward Zn^+2^.

Equilibrium data were fitted with the Langmuir model while the kinetic data were fitted with the pseudo-second-order model following the nonlinear regression with the aid of Origin 2020 software, and the fitting results are shown in Figure 8e,f and Table 3. Figure 8e,f show a good agreement between the experimental data (points) and the models (dashed lines) with the regression parameters shown in Table 3. For the two models and all the samples, the obtained R^2^ was greater than 90, indicating the feasibility of the models used. Langmuir’s model usually suggests monolayer adsorption with a higher chance of chemical rather than physical adsorption. On the other hand, the pseudo-second-order model suggests that each Zn^+2^ occupies two DA groups. This suggests another reason for the low adsorption capacity obtained by SNP-0.5DA and SNP-1.0DA. Since the amount of DA in these samples is lower than in SNP-1.5DA, as suggested by the TGA analysis (Figure 3), the distribution of the DA groups on the surface may not be crowded enough to give two DA groups close enough to hold one Zn^+2^. The Langmuir constant (K_L_) indicates the extent of interaction between the Zn^+2^ and the adsorbents. The K_L_ value of SNP-1.5DA is the highest. This is due to the high content of NH_2_ groups that are accessible to the Zn^+2^_,_ leading to the highest q_m_ value of 136 mgg^−1^. On the other hand, SNP-0.5DA and SNP-1.0DA have lower K_L_ and q_m_ values than those of SNP despite their content of NH_2_. This is mainly due to the restricted accessibility of these functional groups and the loss of surface area as a result of aggregation and pore blockage. The rate constant K_P2_ shows that the fastest kinetics was obtained by SNP-1.0DA followed by SNP-0.5DA. This is mainly because these two samples have no or very limited porosity, and thus all the available adsorption sites are located on the external surface of the samples. This implies that for the Zn^+2^ ions to reach these active sites they have to diffuse in the bulk solution only, while for SNP-1.5DA most of the active sites are located within the porous structure. Accordingly, the Zn^+2^ ions need to diffuse first in the bulk solution, which is usually the fastest step. Then, it will diffuse within the pores to reach the internal NH_2_ groups, and this is the slowest and most controlling step for the adsorption process.

## 4. Conclusions

In this work, amine-modified nano-silica was prepared via a simple one-pot synthesis under very mild conditions. Four samples were prepared by changing the amount of DA precursor added to the synthesis mixture (0, 0.5, 1.0, and 1.5 mL). Among the prepared samples, SNP-1.5DA showed very attractive performance for the adsorption of Zn^+2^ with very high sensitivity and 100% removal from very dilute solutions up to 60 ppm and with relatively fast kinetic. These characteristics, with the good performance and simple and fast synthesis procedure, make this sample a good candidate for commercial applications after a deeper study to scale up the synthesis procedure.

## Figures and Tables

**Table 1:** Synthesis compositions for the prepared nanocomposites.

Sample	Triton X-100 (g)	Methanol (g)	Cyclohexane (g)	TEOS (g)	DA (mL)	H_2_O (g)
SNP	21	18.75	10	2.5	0.0	12.75
SNP-0.5DA	21	18.75	10	2.5	0.5	12.75
SNP-1.0DA	21	18.75	10	2.5	1.0	12.75
SNP-1.5DA	21	18.75	10	2.5	1.5	12.75

**Table 3:** The Langmuir adsorption isotherm model parameters and the pseudo-second-order kinetic model parameters for the prepared samples.

Sample	Langmuir Parameters		
	K_L_ (L.mg^−1^)	q_m_ (mg.g^−1^)	R^2^
SNP	0.135 ± 0.053	102 ± 10	0.942
SNP-0.5DA	0.072 ± 0.034	79 ± 11	0.912
SNP-1.0DA	0.084 ± 0.031	73 ± 6	0.994
SNP-1.5DA	1.573 ± 0.346	136 ± 5	0.986
**Sample**	**Pseudo-second-order parameters**		
	**K_p2_ (g.mg^−1^.min^−1^)**	**q_e_ (mg.g^−1^)**	**R^2^**
SNP	1.467 × 10^−4^ ± 6.769 × 10^−5^	137 ± 21	0.970
SNP-0.5DA	0.025 ± 0.010	28 ± 1	0.973
SNP-1.0DA	0.022 ± 0.010	25 ± 1	0.957
SNP-1.5DA	6.190 × 10^−4^ ± 8.069 × 10^−5^	109 ± 3	0.994

## Data Availability

Not applicable.
